# Updates in the Pharmacologic Prophylaxis and Treatment of Invasive Candidiasis in the Pediatric and Neonatal Intensive Care Units: Updates in the Pharmacologic Prophylaxis

**DOI:** 10.1007/s40506-022-00258-z

**Published:** 2022-05-16

**Authors:** James Hunter Fly, Seerat Kapoor, Kelly Bobo, Jeremy S. Stultz

**Affiliations:** 1 Department of Clinical Pharmacy and Translational Science, University of Tennessee Health Science Center, 881 Madison Ave., Memphis, TN 38163, USA; 2 Department of Pharmacy, Le Bonheur Children’s Hospital, Memphis, TN, USA

**Keywords:** Pediatric, Echinocandin, Fluconazole, Candidiasis, Antifungal, Amphotericin B

## Abstract

**Purpose of review:**

The goal of this review was to provide an update on the prevention and treatment options for invasive candidiasis (IC) in the neonatal intensive care unit (NICU) and pediatric intensive care unit (PICU).

**Recent findings:**

Studies have further validated the use of fluconazole for IC prophylaxis among high-risk patients in the NICU. It remains unclear if prophylaxis leads to resistance development and the ideal dosage regimen is still not clear. Recent studies have been published comparing caspofungin and micafungin to amphotericin B and illustrated similar efficacy outcomes in the NICU. Micafungin now has approval from the United States Food and Drug Administration (FDA) for use in infants < 4 months of age. Prophylactic strategies in the PICU could include zinc and vitamin D. Anidulafungin has recent non-comparative data supporting use in pediatric patients older than 1 month of age and also has a recent FDA approval for use in children 1 month of age and older.

**Summary:**

Fluconazole prophylaxis remains a reasonable strategy in select NICU patients, although further analyses of resistance and the optimal dosage regimen are needed. Echinocandins are potential therapeutic options for non-meningitis or urinary tract infections in both the neonatal and pediatric population.

## Introduction

Invasive candidiasis (IC) is a nosocomial infection that occurs in the neonatal intensive care unit (NICU) and the pediatric intensive care unit (PICU) [1]. While molds like *Aspergillus* also occur, they are much less common than *Candida* species [2]. The most common pathogenic species is *C. albicans*, but non-albicans *Candida* like *C. Parapsilosis*, *C. tropicalis*, and others are frequent and regional differences in pathogenic species have been described [3].

The categorization of “invasive” candidiasis typically includes Candidemia, *Candida* meningitis, *Candida* endocarditis, but usually does not include mucosal candidiasis (e.g., oropharyngeal or vaginal candidiasis) [4]. Unique to the pediatric population, candidiasis of the urinary tract can be associated with poor outcomes and central nervous system involvement is commonly of concern [5]. Analyses of prophylactic strategies may utilize colonization site cultures (e.g., respiratory and gastrointestinal tract) as a surrogate for more severe disease, but treatment approaches generally focus on the less common, but more severe invasive candidiasis.

In 2010, IC was reported as 1.4 infections/1000 NICU admissions overall (down from 3.6 in 1997), with multifold higher rates reported in patients with birth-weights < 1000 g and the highest in those < 750 g (23.8 infections/1000 NICU admissions in 2010, down from 82.7 in 1997) [6]. The reductions up to 2010 were likely due to increased use of fluconazole prophylaxis and decreased antimicrobial utilization, but a recent study has described a leveling off in infection reduction from 2011 to 2018 [6, 7]. In the PICU, rates of IC have been reported as 4.2 infections/100 admissions of patients at least 7 days of age with at least a 3-day admission [8]. A recent analysis reported a reduction in infections in the PICU from 2011 to 2018 [7].

Invasive candidiasis is associated with high morbidity and mortality, with mortality rates ranging from 10 to 28% for pediatric patients as a whole [3]. Mortality rates in the NICU for IC are estimated to be around 20% and are inversely correlated with birth-weight, with reported mortality up to 50% in very low birth-weight infants [3]. Among survivors, long-term neurodevelopmental complications can occur in 10–50% of patients [3, 9]. Among severe sepsis patients in the PICU, a multicenter database analysis reported case fatalities for fungal infections of 25.9% in 2014. This was higher than the 13.9% mortality for *Klebsiella pneumoniae*, which had the next highest mortality among non-viral pathogens [2]. Due to the severe complications associated with IC, effective prevention strategies and optimal treatment strategies are imperative.

The guidelines currently published for the treatment of candidiasis with pediatric recommendations are the European Society for Clinical Microbiology and Infectious Diseases (ESCMID) and Infectious Diseases Society of America (IDSA) guidelines published in 2012 and 2016, respectively [4, 10]. Some of the recommendations in the guidelines were based on minimal evidence and new data has been published since the 2016 update. In this review, we aimed to discuss recent publications and elucidate if or how these publications could impact the prevention and treatment of IC in neonatal and pediatric intensive care units. Table 1 provides a brief summary of the important new literature and the implications of that literature on practice.

## Antifungal utilization in the NICU and PICU

**Fig. 1:**
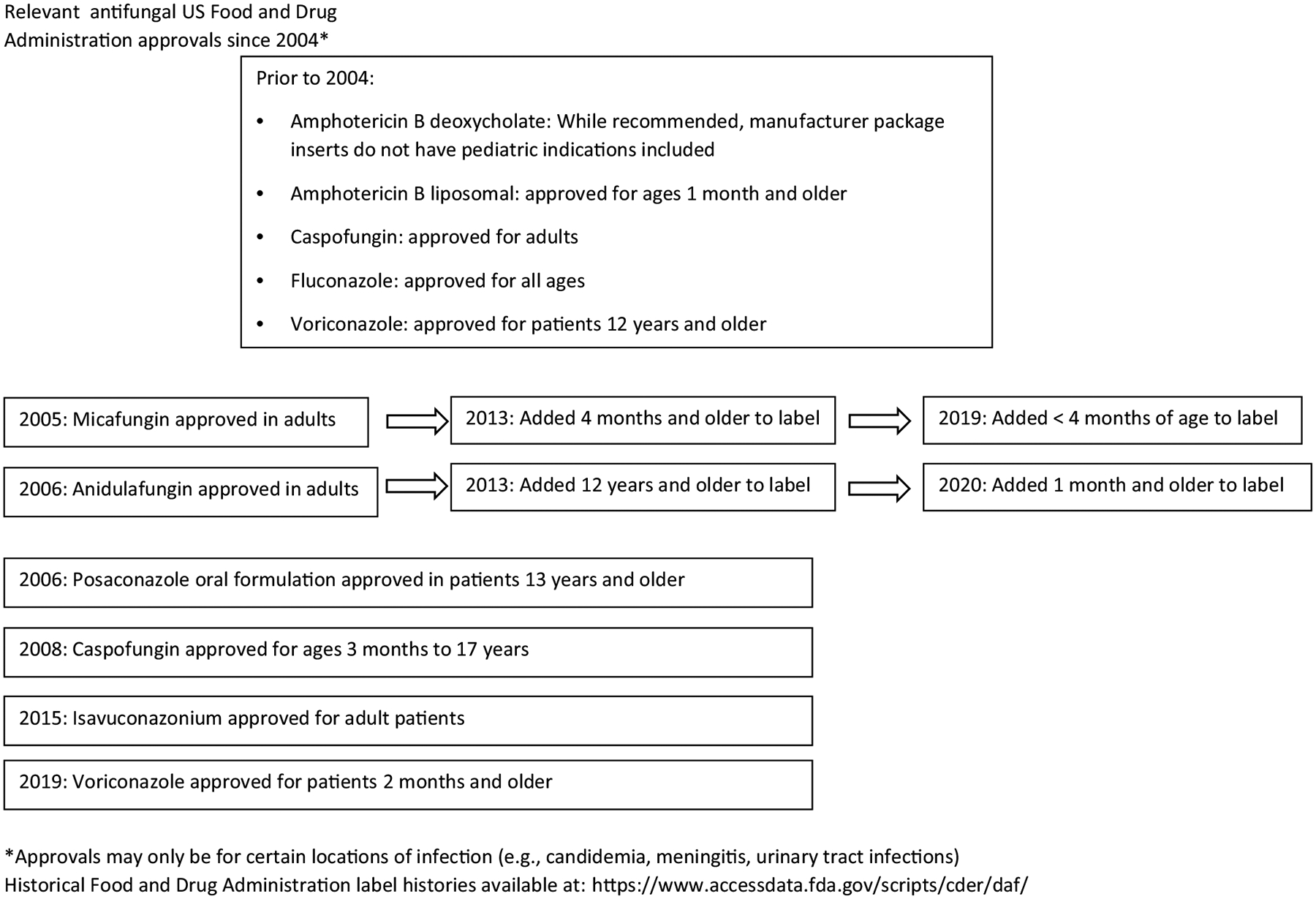
Relevant antifungal US Food and Drug Administration approvals since 2004. Approvals may only be for certain locations of infection (e.g., candidemia, meningitis, urinary tract infections) (Historical Food and Drug Administration label histories available at: https://www.accessdata.fda.gov/scripts/cder/daf/).

As with other medications, antifungals have historically been approved by the Food and Drug Administration (FDA) for use in adults and then studies were done in the pediatric and neonatal population to assess safety, pharmacokinetics, and efficacy. Figure 1 illustrates the dates of pediatric FDA approvals for the systemic antifungals currently available in the United States since 2014. Off-label antifungal use, either with off-label indications or age groups, is common in pediatric and NICU patients, especially for salvage therapy. For example, amphotericin B deoxycholate is a guideline-recommended treatment option that has been used for years, but current manufacturer package inserts do not have data for use in pediatric patients [11].

Fluconazole is the most common antifungal used in the neonatal population, with most of its utilization attributed to prophylaxis that may not be consistent with guideline recommendations [12, 13]. The second highest antifungal utilized in the NICU population is amphotericin B, which comes in the “conventional” or deoxycholate form (AmpB-D), a liposomal form (AmpB-L), and a lipid complex form. Other agents like voriconazole (available intravenously or orally), posaconazole (available intravenously or orally), itraconazole, and recently approved isavuconazonium account for a small proportion of utilization. Importantly, most utilization studies were completed before some of these agents became available or labeled in the pediatric population and thus utilization may have recently changed.

Antifungal utilization data specific to the PICU are difficult to discern, but in the pediatric population as a whole, fluconazole use still predominates, followed by echinocandins and voriconazole [12]. Importantly, echinocandins and voriconazole are used for prophylaxis in the hematologic/oncologic population and this may account for the bulk of their use in the PICU. Amphotericin B products have significantly less use. Other antifungals are used in this population, but still less compared to fluconazole and echinocandins. Antifungal drug information and clinical pearls are presented in Table 2.

## Guideline recommendations for antifungal prophylaxis in the NICU

The most recent 2016 guidelines suggest that antifungal prophylaxis in the NICU can be effective at preventing IC and potentially mortality. The recommended regimen is a 3–6 mg/kg twice weekly dose of fluconazole for 6 weeks only in patients < 1000 g in institutions with a high IC incidence rate (> 10%) [4].

These recommendations were based on multiple randomized control trials and observational studies illustrating general benefit in this high-risk population without apparent risks or resistance described. In Cochrane Database meta-analyses prior to the 2016 guideline, antifungal prophylaxis (primarily fluconazole) decreased IC and studies with higher initial rates of fungal infection (typically cited as > 10%) were more likely to have an impact [14, 15]. The commonly cited number needed to treat for benefit (NNTB) is approximately 11 in the NICU population based on an aggregate incidence of ~ 16% in the control groups, but the NNTB would vary based on the initial institutional incidence rate. The meta-analyses prior to the 2016 guideline publication did not illustrate a significant reduction in mortality [14, 15]. Institutions have also used more selective criteria such as patients < 750 g with central lines or patients < 1500 g that required more than 3 days of broad-spectrum antibiotics and still showed a successful reduction in infections without a subsequent increase in IC in patients not receiving prophylaxis [16].

## Recent publications related to antifungal prophylaxis in the NICU

Since the publication of the 2016 guidelines, one pre-post implementation study failed to find a reduction in IC when fluconazole 3 mg/kg/day twice weekly was used for 4 weeks in patients born < 1000 g at an institution with a baseline IC rate of 4.4% [17]. Two meta-analyses since the last guidelines, which combined studies with different dosing schedules, were able to show that overall fluconazole prophylaxis decreased IC-related mortality [18, 19]. These new data are generally confirmatory of previous recommendations for fluconazole prophylaxis at institutions with high IC rates in high-risk patients.

New literature and secondary analyses of previous studies have attempted to determine an optimal fluconazole prophylaxis dosing regimen, although it is still controversial. Adult based prophylactic studies have suggested that concentrations > 2 mcg/mL (typically an AUC of 50 mcg*h/L) are ideal for prophylaxis. Some *Candida* species have minimum inhibitory concentrations of 4 mcg/mL. Two population pharmacokinetic monitoring studies have illustrated that 3 mg/kg twice weekly would achieve troughs > MIC for most *Candida* species, but 6 mg/kg twice weekly may be needed for *Candida* with an MIC of 4 mcg/mL or above [20, 21]. Common *Candida* MICs at a given institution should be taken into account when deciding on an institutional fluconazole prophylaxis regimen.

When looking at clinical outcomes based on dosage, one meta-analysis suggested that there was no difference in IC or overall mortality when comparing dosing regimens of 3, 4, or 6 mg/kg/dose administered twice weekly or every 3 days, although 6 mg/kg/dose was considered better for the mortality benefit on sensitivity analyses performed by the authors [19]. The authors overall recommended 3 mg/kg/dose as the best dose to minimize exposure but still have an equal effect. A second meta-analysis from 2021, also including multiple dosing regimens, found that the mortality benefit seen in the overall cohort was seen primarily in studies using the dosing regimen of 3 mg/kg/day every 3 days weeks 1 and 2 and increased over the next 3 weeks to daily dosing during weeks 5 and 6 [18]. The ideal fluconazole prophylactic dosing regimen remains unclear.

A large concern surrounding fluconazole prophylaxis is the potential for resistance development and recent studies have attempted to answer this question. A secondary analysis of a randomized study on fluconazole prophylaxis looked specifically at resistance development and found a higher MIC among *Candida* colonization cultures after prophylaxis, although this MIC was still in the susceptible range (median MIC of 1 mcg/mL) [22•]. Most of the colonizing *Candida* obtained were *C. albicans* with *C. parapsilosis* being the second most common. There were also no breakthrough infections with fluconazole-resistant *Candida* in this study. In a secondary analysis of a prospective observational study regarding fungal infections in the NICU, none of the three infants with resistant *Candida* isolates (2 *C. albicans* and 1 *C. glabrata* out of a 110 patients) received fluconazole prophylaxis prior to the infection [23]. A recent pre-post implementation study from Korea reported a non-significant increase in resistance within patients using a 3 mg/kg/dose twice weekly in fluconazole-resistant *C. Parapsilosis* (0/3 resistant in the control group and 5/9 in the fluconazole group [17]. A randomized controlled trial from India also reported no *C. albicans* infections with fluconazole resistance when prophylaxis was used, but 60% resistance among *C. tropicalis* (a species not commonly seen in other studies) [24]. An additional study from Taiwan also illustrated the potential for *M. furfur* colonization with 1 breakthrough infection in patients receiving prophylaxis versus minimal colonization and no infections in patients without prophylaxis [25]. Multiple studies did not find evidence of future resistance problems with fluconazole prophylaxis, but regional differences in fungal infection pathogens and low sample sizes preclude an accurate determination of the occurrence of fluconazole resistance due to prophylaxis. Additionally, studies have not looked at the impact of fluconazole prophylaxis on institutional or regional susceptibility patterns for *Candida* species.

## Guideline recommended treatment options for invasive candidiasis in the NICU

The 2016 IDSA and 2012 ESCMID guidelines both recommend either fluconazole (if no prior fluconazole exposure) or amphotericin B for neonatal IC treatment, with the IDSA guidelines recommending caution with the use of the lipid/liposomal amphotericin B formulations in the neonatal population [4, 10]. These recommendations were based on small prospective studies including 23 and 56 infants and a retrospective database study. The first study compared AmpB-D and fluconazole (23 total patients) and found similar treatment outcomes with a better safety profile in the fluconazole cohort [26]. The second study compared AmpB-D, AmpB-L, and amphotericin B colloidal dispersion (an amphotericin B dosage form not currently being manufactured in the USA) to treat bloodstream infections and found all formulations to be effective (56 total patients) [27]. This study did not evaluate central nervous system (CNS) infections, a common concern in neonates. A database study found that among 730 patients with IC (65% with blood involvement, 21% urine involvement, < 1% CNS, and 14% mixed), infants treated with lipid formulations of Amphotericin B (including liposomal, lipid complex, and colloidal dispersion) had higher mortality rates than infants treated with fluconazole or AmpB-D after controlling for other factors. Overall group mortality was 19%, but was 29% in the AmpB lipid-treated patients [28].

Prior to the 2016 guideline publication, there was limited data for echinocandins in neonates. Micafungin was compared to AmpB-L in a prospective study containing 14 patients where 7/7 (100%) patients in the micafungin group vs 4/7 (57.1%) in the AmpB-L group had treatment success [29]. Caspofungin was compared to AmpB-D in 32 neonates with IC and demonstrated a favorable response in 86.7% of patients compared to 41.7% (*p*-value = 0.04) in the AmpB-D cohort [30]. Of note, only a total of 5 patients from both these studies in the echinocandian cohorts had CNS infections, and echinocandins are reported to have poor penetration of the CNS (Table 2). There is a concern for nephrotoxicity when using AmpB-D; however, studies in the neonatal population have an acceptable toxicity profile with AmpB-D [27, 28]. Fluconazole is traditionally well tolerated, but has intrinsic resistance to *C. krusei* and *auris* and can have variable resistance to *C. glabrata* and *tropicalis* [31, 32]. With a continuing concern for resistance development over time, it is important to evaluate alternative treatment options.

## Recent publications related to treatment options for invasive candidiasis in the NICU

Micafungin gained FDA approval for patients under 4 months of age in 2019 based on safety data from 168 patients accrued from 9 clinical trials (Fig. 1). Although the label does include descriptions of 10 mg/kg/day or higher dosing specifically for patients with meningoencephalitis, the 4 mg/kg/day dose was the approved dose and it is not indicated for patients with meningoencephalitis or ocular disease [33]. Caspofungin does not have FDA approval for use in neonates/preterm infants < 3 months of age, and anidulafungin has approval for patients 1 month of age and older [34, 35]. Recent prospective studies have investigated the safety and efficacy of echinocandins as compared to the traditional standard of care, AmpB-D, although both were terminated early due to slow recruitment [36••, 37••].

A phase 3, randomized, double-blinded, parallel group, non-inferiority study randomized infants from 3 to 120 days of life with a positive *Candida* sp. culture to receive either micafungin 10 mg/kg/day or AmpB-D 1 mg/kg/day [36••]. There were 20 patients included in the micafungin group (16 with candidemia and 7 with urinary tract involvement) and 10 in the AmpB-D group (7 with candidemia and 1 with urinary tract involvement). Two patients in the micafungin and one in the AmpB-D arm had CNS involvement identified by the data review board. Fungal-free survival (FFS) was achieved in 12 patients (60%) in the micafungin group compared to 7 (70%) in the AmpB-D group. In a secondary analysis, there was a positive clinical response on day 7 in 61% and 70% in the micafungin and AmpB-D groups. Persistent fungal infections occurred in 2 (10%) of infants in the micafungin group and 2 (20%) of the AmpB-D group, with *C. parapsilosis*, *glabrata*, and *albicans* being responsible. Adverse events occurred in similar proportions for each group, with 18 (90%) in the micafungin group and 9 (90%) in the AmpB-D group experiencing a treatment-emergent adverse event. Bilirubin and hepatic enzymes were elevated more in the micafungin group and electrolyte replacement and acute kidney injury (AKI) were higher in the AmpB-D group. A pharmacokinetic analysis based on targets from animal models of *Candida* meningoencephalitis, demonstrated a dose of 10 mg/kg/day of micafungin is adequate for CNS disease while also being safely tolerated [36••].

A phase 2 randomized, multicenter, double-blind, comparator-controlled study also compared caspofungin 2 mg/kg/day to AmpB-D 1 mg/kg/day [37••]. The study enrolled 33 patients (1 with meningoencephalitis, 24 with candidemia) in the caspofungin arm and 16 in the AmpB-D arm (1 with meningoencephalitis, 8 with candidemia). FFS, 71% and 68.8%, respectively, was similar between the 2 groups. Two (6.5%) patients in the caspofungin group had drug-related side effects versus 2 (12.5%) in the AmpB-D arm [37••].

A multicenter, observational, prospective trial published in 2019 from France investigated the safety and efficacy of micafungin in pediatric patients [38]. This study enrolled a total of 29 non-hematologic neonates treated for mostly probably IC, with only two neonates having confirmed IC (*C. albicans* and *C. glabrata*). The investigators found micafungin to be effective in 28/29 (97%) of infants. The safety analysis in NICU patients found 4 adverse events and none were considered to be related to the study drug. The mean dose of micafungin in this study was 7.6 mg/kg/day. The authors concluded that micafungin was well tolerated in neonates.

A secondary analysis of a prospective observational study described the prevalence of *Candida* sp. resistant to AmpB-D, fluconazole, and micafungin in infants with IC [23]. There were 110 infants that yielded a total of 308 *Candida* isolates with susceptibility data. The most common *Candida* sp. were *albicans* (60%), *parapsilosis* (35%), and *glabrata* (3%). All isolates were found to be susceptible to AmpB-D and micafungin; 3 were resistant to fluconazole. There were 42% of included infants with a high MIC pathogen (defined above the 90th percentile MIC of all isolates) to one of the three aforementioned agents. Having this high MIC pathogen did not correlate with prior receipt of fluconazole or nystatin prophylaxis and did not correlate with death rates, neurodevelopment impairment, or a combined endpoint at 18–22 months of age. While a subgroup analysis was not performed, it should be noted that all of the 7 patients with a *Candida* MIC of ≥ 2 mcg/mL for fluconazole either died or had neurodevelopmental impairment at 18–22 months. The study authors concluded MIC elevations that are still considered susceptible may not impact treatment outcomes, but the number of resistant isolates was small. Thus, outcome differences and treatment options for patients harboring a *Candida* sp. with a fluconazole MIC ≥ 2 mcg/mL remain unclear.

The literature surrounding the safety and effectiveness of echinocandin use in the NICU has increased since the publication of the 2016 guidelines and may suggest the use of this class of antifungal agents in select patient populations, although efficacy is still unclear in CNS disease. Alternative azole antifungals aside from fluconazole have been used for salvage therapy or non-candidal fungal infections (e.g., voriconazole for *Aspergillus*), but robust safety and efficacy data in the neonatal population is very limited (Table 2).

## Guideline recommended fungal prophylaxis in the pediatric intensive care unit

In the 2016 guidelines for Adult ICUs, prophylaxis could be used in centers with > 5% IC rates in select high-risk patients. This verbiage does not specifically state use in a PICU, although some institutions may use this as guidance to determine if prophylaxis may be needed [4]. Risk factors for IC in the PICU identified via multivariable prediction models have included length of PICU stay, receipt of parenteral nutrition, need for central venous catheters, malignancy, thrombocytopenia, and broad-spectrum antibiotic use (specifically vancomycin and anaerobic active agents for > 3 days) [8, 39]. Utilizing a combination of factors likely increases the sensitivity and specificity of these predictive factors. The ideal risk factor or combination of risk factors for IC has not been clearly elucidated, and thus, it can be difficult to determine the high-risk patients who need prophylaxis and/or treatment initiation [40]. It is important to note that many immunosuppressed patients or patients with chronic diseases predisposing them to infections (e.g., malignancy, chronic granulomatous disease, solid organ, or bone marrow transplant) may be receiving prophylactic antifungals as part of management for their primary disease. These antifungals are usually continued while in the PICU.

There is also no preferred agent for prophylaxis in PICU if it is to be started [40]. In the 2016 guidelines for adult ICUs, fluconazole is offered as a possible option with echinocandins as an alternative [4]. There is a concern for increases in resistance and adverse events, and therefore, prophylaxis with non-antifungal alternatives is commonly considered [41]. A prophylaxis method recommended in the 2016 guidelines for adult ICUs is daily chlorhexidine baths to reduce the incidence of bacteremia and as an end result also potentially reduce candidiasis, but other methods of prevention are critical to study to help prevent candidiasis in the PICU [42].

## Recent publications regarding prophylaxis in the PICU

Recent studies have investigated the use of additional non-antifungal prophylaxis strategies to prevent and reduce the resistance of antifungal drugs. A randomized, placebo-controlled trial including 724 children (1–5 years old) assessed the efficacy of 20 mg elemental daily zinc (administered as a syrup) supplementation in patients admitted to the PICU. Plasma zinc concentrations are often low in critically ill patients and zinc has shown antimicrobial efficacy against *C. albicans*. Candidemia occurred in 10 patients (2.8%) with zinc supplementation compared to 22 (6%) in the placebo group (*p* = 0.03) [43•].

The same authors also studied the use of 300 IU vitamin D supplemented yogurt drinks as an alternative prophylaxis measure for 416 PICU patients (1–5 years of age) receiving broad-spectrum antibiotics for > 48 h. *Candida* colonization, especially in the gastrointestinal tract, is believed to correlate with higher incidences of IC and in vitro studies suggest vitamin D has fungicidal activity against *C. albicans*. Outcomes were measured 14 days after initiation. Five patients (0.5%) in the vitamin D group had candidemia versus 14 (6.7%) in the placebo group (*p* = 0.02) [44]. These two strategies may be simple non-antifungal-based methods to help decrease IC in the PICU.

## Guideline recommendations for treatment of invasive candidiasis in the PICU

A wide array of patients are admitted to the PICU, including neonates and some adults with chronic childhood diseases. In general, patients are admitted to the PICU for advanced life support such as intubation due to respiratory failure, vasopressor administration, or other forms of advanced life support for hemodynamic compromise. Thus, antifungal use in the PICU is likely focused on the treatment of candidal sepsis.

Prior to the publication of the 2016 guidelines, caspofungin and micafungin had FDA approval for use in infants and children, but anidulafungin only had approval for patients > 16 years of age (Fig. 1). Although the PICU population is not clearly defined in the 2016 candidiasis guidelines, the guidelines recommend echinocandins for first line for treatment of IC in nonneutropenic and neutropenic patients and thus would likely be considered first line in the PICU. Fluconazole is an option if the patient is not severely ill and as step-down therapy. Amphotericin B products and voriconazole are typically reserved for specific non-*Candida* infections or if there is resistance [4]. Table 2 provides additional information regarding antifungals in the pediatric population.

## Recent publications related to the treatment of invasive candidiasis in the PICU

Two recent meta-analyses including pediatric and some neonatal studies illustrated similar outcomes between echinocandins and amphotericin B products possibly with less discontinuation of echinocandins due to side effects [45, 46]. A multicenter, observational, prospective trial from France included 14 PICU patients treated for potential IC (5 with proven IC) and illustrated effective outcomes in 12/14 patients (85.7%). One patient had hepatic lesions which were deemed to have a possible relation to micafungin [38]. Significant new comparative publications were not identified since the publication of the 2016 guidelines.

Anidulafungin received FDA approval in 2020 for patients aged 1 month and up for candidemia or intra-abdominal abscesses/peritonitis due to *Candida* species, but not endocarditis, osteomyelitis, or meningitis (Fig. 1). This labeling came from 2 open-label non-comparative studies involving 49 patients 2–18 years and 19 patients 1 month to 2 years and [47, 48••]. The study with patients 2–18 years had an all-cause mortality rate of 14.3%, although only one death was likely due to IC with an overall global success rate of 70.8%. Bacteremia was the most common IC (93.4% of the study population) [47]. In the study with infants 1 month–2 years of age, 16 had culture-confirmed IC with 15/16 having candidemia and one infection involving the urinary tract. Only one (5.3%) patient died during the study and it was 40 days after enrollment. Global response success occurred in 11 patients (68.8%) [48••]. The authors concluded pediatric patients treated with anidulafungin had a similar response to adults and pharmacokinetic analyses achieved a similar drug response compared to adults. Due to the lack of a comparative study, the labeling change primarily extrapolated efficacy data from adult studies.

Overall the new data is not likely to significantly change IC treatment options in the PICU. It is important to remember that PICU patients could have renal or hepatic dysfunction and may require advanced life support techniques, continuous renal replacement therapy (CRRT), and/or extracorporeal membrane oxygenation (ECMO), all of which may impact antifungal drug dosing and selection. For example, patients in acute renal failure need a dose reduction for fluconazole use since it is excreted largely unchanged in the urine. Conversely, larger doses are needed in CRRT and ECMO due to increased fluconazole elimination and increased volume of distribution, respectively [49]. In these patients, the dosage and selection of antifungals should be individualized based on pharmacokinetic and safety considerations of both the drug and the patient.

## Summary

Invasive candidiasis is a concerning cause of infections in the NICU and PICU. Prophylaxis in NICU patients can be considered based on institutional IC rates and patient characteristics. The available evidence still has not clearly illustrated detrimental resistance development to this strategy. But, more data on the occurrence and relevance of antifungal resistance in institutions utilizing prophylaxis is needed. There are recent, albeit small sample size, studies utilizing echinocandins that could impact the initial antifungal treatment choice for IC in the NICU, and micafungin is now FDA approved for use in patients less than 4 months of age. It remains difficult to determine what PICU patients would benefit from antifungal prophylaxis, and strategies aside from antifungal prophylaxis could be considered. Echinocandins likely remain the primary treatment option for IC in PICU patients.

## Figures and Tables

**Table 1:** Summary of new data since publication of the previous guideline for antifungal use in the NICU and PICU

Patient population	Recommendations—last guideline update	Major new data summary	Potential practice implications	Unanswered questions
Fluconazole prophylaxis in the NICU or neonates	IDSA: fluconazole twice weekly if the patient is < 1000 g and institution has > 10% IC ESCMID: same as IDSA, but could consider risk stratification in centers with low incidence	Evidence does not clearly indicate the development of resistant *C. albicans*, but *C. tropicalis* has reports of increasing resistance Colonization of *M. furfura* may be impacted	Prophylaxis is still a reasonable option in select highest risk patients and/or at centers with high rates of infections	Long-term safety of antifungal prophylaxis Efficacy of non-fluconazole prophylaxis Potential for resistance still not definitive Dosing unclear
Candidiasis treatment in the NICU	IDSA: amphotericin B deoxycholate (precautionary use of lipid products) or fluconazole; echinocandins as salvage therapy ESCMID: amphotericin B deoxycholate or lipid products or fluconazole	Micafungin or caspofungin may have similar efficacy compared to amphotericin B deoxycholate Micafungin now FDA approved for infants less than 4 months Resistance is uncommon, and MIC differences within the susceptible rage may not predict efficacy	Echinocandins may be an alternative first-line agent Echinocandins are still not well studied for neonatal CNS and urinary infections	Ideal amphotericin product Comparison of echinocandins to fluconazole Use of echinocandins or liposomal amphotericin in CNS/urinary diseases
Antifungal prophylaxis in the PICU	IDSA: could be considered in ICUs at institutions with high rates of IC (>5%) ESCMID: no evidence-based recommended, but fluconazole could be considered in high-risk patients (e.g., abdominal surgery)	Non-fluconazole prophylactic strategies like zinc and vitamin D may have a role Select populations in the PICU may receive various prophylactic regimens due to their underlying conditions	Consider ensuring receipt of zinc and vitamin D in critically ill PICU patients with risk factors for IC Target patients primarily based on their underlying disease state	Should prophylaxis be used at all in patients lacking other risk factors Should primarily non-fungal prophylaxis be used (e.g., bundles)
Candidiasis treatment in the PICU	IDSA and ESCMID: echinocandins preferred first line in patients over 3–4 months of age for IC treatment	Minimal new data updates for treatment Anidulafungin received FDA approval in patients 1 month and older	Continue with prior guideline recommendations	What dosages are best to use in critically ill patients

*NICU*, neonatal intensive care unit; *PICU*, pediatric intensive care unit; *IDSA*, Infectious Diseases Society of America; *IC*, invasive candidiasis: *ESCMID*, European Society for Clinical Microbiology and Infectious Diseases; *CNS*, central nervous system; *FDA*, United States Food and Drug Administration; *PICU*, pediatric intensive care unit

**Table 2:** Pertinent antifungal dosing, clinical pearls, and potential places in therapy

Drug	Doses utilized	Notable PK parameters	Likely place in therapy	Other Pearls
Amphotericin B deoxycholate IV [50–53]	*Neonatal*: 1 mg/kg qday *Children*: 0.25–0.5 mg/kg, can be titrated to 1.5 mg/kg qday	Vd: 0.38–3.99 L/kg Neonatal CNS penetration reported to be 40–90%^13^ Children CNS penetration is poor Excretion: urinary T ½: 14.8 (5 to 82 h) in neonates 11.9–48 h in children and adults	Invasive candidiasis in the neonatal population	Can give a test dose prior to administration Infusion-related reactions are common. Pre-medicate with an NSAID/acetaminophen ± diphenhydramine or hydrocortisone mono-therapy 30 to 60 min prior to drug administration. Rigors can be treated with meperidine Nephrotoxicity can occur during treatment. Adequate hydration and monitoring of kidney function are required. **Neonates are reported to have less nephrotoxicity than adults**
Amphotericin B (liposomal) IV [53–55]	*Neonatal*: 3–5 mg/kg qday (NON CNS) *Children*: 3–5 mg/kg qday	Vd: 0.1–0.16 L/kg CNS penetration: 1–3% Excretion: urine and feces T ½: 6–23 h	Resistant *Candida* infections Empiric therapy for neutropenic patients Invasive *Aspergillus*	Drug can have increased terminal half-life due to slow redistribution from tissues Dosing is not interchangeable between traditional and liposomal product Less infusion reactions and nephrotoxicity compared to traditional amphotericin
Fluconazole IV, tablet, suspension [10, 21, 56–58]	*Neonatal*: treatment: 12–25 mg/kg loading dose, then 6–12 mg/kg qday Prophylaxis: 3–6 mg/kg/dose twice weekly *Children*: 6–12 mg/kg loading dose 3–12 mg/kg qday	Vd: 0.913 L/kg CNS penetration: 50–90% Excretion: urine T ½: 20–50 h (dependent upon renal function)	Prophylaxis Invasive candidiasis Meningitis Urinary tract infections	Inhibits CYP 3A4, 2C9, and 2C19 enzymes *Candida krusei* demonstrates intrinsic resistance Can cause QT prolongation
Posaconazole IV, delayed-release tablet, and suspension [59, 60]	**All data limited** Oral suspension: infants ≥ 6 months to < 2 years: initial dosing: 200 mg QID 2–6 years: initial dosing: 200 mg QID Children 7–12 years: initial dosing: 300 mg/dose QID Oral delayed-release tablets: children 7–12 years: initial dosing: 200 mg/dose TID IV: **VERY LMITED DATA** Children ≤ 11 years: 6–10 mg/kg BID on day 1, followed by 6–10 mg/kg daily (maximum dose 300 mg)	Vd: oral: 287 L; IV: 261 L CNS penetration: limited data, thought to be poor Excretion: feces 71%, 13% urine T ½: suspension: ~35 h Tablets: 26 to 31 h IV: ~ 27 h	*Aspergillus* prophylaxis Maintenance and salvage therapy for mucormycosis	Oral suspension has better absorption with a high-fat meal or if taken with an acidic beverage Displays saturable absorption Tablet has more predictable absorption than liquid suspension Trough goals are typically > 0.7 mg/L for prophylaxis and 1 mg/L for treatment Concentration recommended to be drawn on day 7 of therapy Inhibits the CYP 3A4 enzyme
Vonconazole IV, tablet, and suspension [60–63]	*Neonatal*: **Limited data case reports:** IV: 12 to 20 mg/kg/day divided every 8 to 12 h *Children 2 to < 12 years*: IV: 9 mg/kg q12 h for 2 doses on day 1 followed by IV: 8 mg/kg/dose every 12 h or PO suspension 9 mg/kg q12 h *Children* ≥ *12 to* ≤ *14 years*: < 50 kg: IV loading dose:9 mg/kg/dose q12 h for 2 doses; followed by 4 to 8 mg/kg q12 h ≥ 50 kg: IV loading dose: 6 mg/kg q12 h for 2 doses followed by 3 to 4 mg/kg q12 h Oral maintenances: < 50 kg: 9 mg/kg/q12 h ≥ 50 kg: 200 mg every 12 h *Adolescents* ≥ *15 years*: IV: loading dose: 6 mg/kg q12 h for 2 doses; followed by 3 to 4 mg/kg q12 h Oral maintenance: < 40 kg: 100 mg q12 h ≥ 40 kg: 200 mg q12 h	Vd: Diphasic in children 2 to < 12 years 0.81 mL/kg (central) 2.2 mL/kg (peripheral)^12^ CNS penetration: 68–100% Non-linear kinetics (most likely Michaelis-Menten) Adults: 4.6 L/kg Excretion: urine T ½: dose dependent, steady state reached on day 3 with loading dose and by day 5–8 without	Invasive *Aspergillus* Salvage therapy for mold infections	Can cause photopsia, neurotoxicity, rash, QT prolongation, hepatotoxicity, and nephrotoxicity (IV product has a cyclodextrin solvent, albeit debated toxicity) Oral formulations have erratic absorption and need to be separated from food. Switch to oral only recommended after significant clinical improvement Trough level goals of 1–5.5 mg/L. Levels of 2–6 mg/L can be targeted if CNS involvement Dose adjustments are non-linear, meaning changes in trough/AUC may not be proportional to changes in dosage Is a substrate for and inhibits CYP 3A4, 2C9, and 2C19 enzymes
Amdulafungin IV [60, 61, 64, 65]	*Neonatal*: Limited data based off of pharmacokinetic study IV: 3 mg/kg once on day 1, then 1.5 mg/kg qday^15^ *Children*: IV: 1.5–3 mg/kg once on day 1, then 0.75–1.5 mg/kg qday (max 200 mg)	Vd: 30 to 50 L CNS penetration: Negligible Excretion: primarily feces T ½: 40 to 50 h	Invasive candidiasis	Monitor liver function tests Not routinely used for urinary tract infections
Caspofungin IV [60, 61]	*Neonatal*: Limited data IV: 25 mg/m^2^ qday or 2 mg/kg qday *Children*: 70 mg/m^2^ qday on day 1, then 50 mg/m^2^ qday	Vd: unknown, non-linear kinetics CNS penetration: negligible Excretion: 41% urine; feces 35% T ½: terminal 40–50 h	Systemic candidiasis Salvage therapy for *Aspergillus*	AUC increased with either renal or hepatic impairment Drug accumulates due to non-linear kinetics Can cause chills and hypotension on infusion Monitor Liver function tests periodically
Micafungin IV [36••, 60, 61, 66–68]	*Neonatal*: 4–10 mg/kg qday *Children*: IV: 2–3 mg/kg qday	Vd: highly variable with age CNS penetration: negligible Primarily hepatic metabolism Excretion: primarily feces T ½ 6.7–21 h	Systemic candidiasis Prophylaxis Salvage therapy for *Aspergillus*	Monitor liver function tests Not routinely used for urinary tract infections Higher doses are often needed for neonates and younger children due to pharmacokinetic differences Higher doses are needed for CNS infections
